# Patient-centered psoriatic arthritis (PsA) activity assessment by Stockerau Activity Score for Psoriatic Arthritis (SASPA)

**DOI:** 10.1186/s12891-015-0512-7

**Published:** 2015-04-01

**Authors:** Burkhard F Leeb, Pia M Haindl, Hans-Peter Brezinschek, Harsono T H Mai, Christoph Deutsch, Bernhard Rintelen

**Affiliations:** 2nd Department of Medicine, Center for Rheumatology, Lower Austria, State Hospital Stockerau, Karl Landsteiner Institute for Clinical Rheumatology, Landstrasse 18, A-2000 Stockerau, Austria; Private Rheumatology Office, Babogasse 20, A-2020 Hollabrunn, Austria; Department for Rheumatology and Immunology, Medical University of Graz, Auenbruggerplatz 2, A-8036 Graz, Austria

**Keywords:** Psoriatic arthritis, Disease activity assessment, Patient questionnaire

## Abstract

**Background:**

To investigate whether a modified Rheumatoid Arthritis Disease Activity Index-5 could be applied as a routine assessment tool for psoriatic arthritis (PsA) patients.

**Methods:**

Ninety-seven PsA outpatients (mean age 49.78 years; age range 23–80 years; 49 male, 48 female), completed a prototype questionnaire. Tender and swollen joint counts, including enthesiopathy, physician’s assessment of disease activity on a visual analog scale (MDglob), erythrocyte sedimentation rate, and patient satisfaction with disease status (PatSat: 1 = excellent to 5 = unsatisfactory) were recorded. Factorial analysis was performed and alpha, as a measure of reliability, and tau were calculated. The ultimate five-item questionnaire, calculated by (Q1 + Q2 + Q3 + Q4 + Q5)/5, was then handed over to 152 PsA outpatients (mean age 54.02 years; age range 26–80 years; 82 male, 70 female), and analyzed accordingly.

**Results:**

Analyzing the internal consistency of the prototype questionnaire revealed the highest alpha value of 0.849, on deleting the question targeting disease course. Alpha for the final Stockerau Activity Score for Psoriatic Arthritis (SASPA) was 0.875, with all items contributing to the final result (item loading from 0.573 to 0.910). Kendall’s tau for the relationship between SASPA scores and swollen joint count, tender joint count, and MDglob was 0.34, 0.416, and 0.392, respectively. The sensitivity of the questionnaire to change was demonstrated in patients starting treatment with a tumor necrosis factor blocker (standardized mean difference: 2.1).

**Conclusion:**

The SASPA questionnaire constitutes a fully patient-administered tool to monitor PsA activity. Its reliability, convergent validity, and sensitivity to change were demonstrated.

## Background

Psoriatic arthritis (PsA) frequently joint causes destruction and functional impairment, although its course is generally less aggressive than that of rheumatoid arthritis (RA) [1]. Peripheral joint damage is significantly greater in RA than in PsA after an equivalent duration, however, joint function and quality of life scores frequently indicate the same amount of impairment for both diseases, which may be due to the additional burden of skin disease in PsA [2].

Hence, in some patients, PsA management requires treatment regimens comparable with those of RA [3]. Given the recent developments for the treatment of PsA, remission and minimal disease activity are postulated as treatment goals and may represent a measure to compare therapies [4]. The treat-to-target approach claims to have a better patient outcome [5], which is currently the object of clinical trials [6]. It is one of the overarching principles of this approach that therapy should be based on a shared decision between patient and rheumatologist [5].

Several composite activity scores for PsA have been published, however, because of a lack of consensus; they are not widely utilized [7]. The Disease Activity Score (DAS28-ESR and DAS28-CRP), Simplified Disease Activity Index (SDAI), Composite Psoriatic Disease Activity Index (CPDAI), disease activity in psoriatic arthritis (DAPSA), and Psoriatic Arthritis Disease Activity Score (PASDAS) have been proposed [8-12]. Although validated only for RA, DAS28 and the original DAS (also including the feet) have already been used in PsA clinical trials as secondary efficacy criteria [8,13]. However, DAS28 appears to be explicitly different statistically when applied to PsA patients [14].

Recently, good concurrent validity and discriminant capacity of these disease activity indices were observed, but the proportions of patients classified at the disease activity levels differed. In particular, the number of patients in remission clearly differed among the respective indices [7], indicating that the interchangeability of those indexes is limited.

All those indexes are appropriate for comparisons at the group level, but may not be fully suitable for assessing individual patients. It is crucial for personalized daily patient management, particularly when following the treat-to-target approach [5], to be aware of the patient’s view at any time.

Also, several response criteria, mainly the Psoriatic Arthritis Response Criteria (PsARC) or the modified American College of Rheumatology (ACR) response criteria, have been proposed and subsequently applied in clinical trials to record disease activity and therapeutic efficacy, respectively [15,16]. These tools give relative results with respect to a baseline observation, which, as in routine RA management, can be regarded as an essential reason why those criteria are considered not to meet the needs of daily rheumatologic practice.

Within the field of RA monitoring it is accepted that patient questionnaires for functional status could be more informative when pertaining to prognosis than even a full joint count [17]. It was shown that the three patient-only parameters in the ACR response criteria, namely physical function, pain and global status, were as reliable as the whole core set for describing RA activity changes, and could constitute the basis for therapeutic decisions [18]. In addition, an index of those three self-report scales on the ACR core data set can discriminate between active and placebo treatments in clinical trials, as well as the DAS score [19].

We have demonstrated that a modified version of the Rheumatoid Arthritis Disease Activity Index (RADAI), RADAI-5, refraining from joint counts, can measure RA activity in daily routine with high reliability and proven convergent validity [20]. The time required for RADAI-5 is <30 sec. This enhances its feasibility, which apart from reliability and validity is the most important prerequisite for daily routine application [21].

Keeping in mind the dilemma with accurate monitoring of patients, it 12891_2015_512med reasonable for us to transfer the concept of the RADAI-5 to PsA. The ultimate intention was to devise a fully patient-administered tool to measure PsA activity in daily routine.

## Methods

### Prototype questionnaire

RADAI-5 was established in the German language and comprises five items in a Likert format from 0 to 10 [22]. The respective questions are “How active was your arthritis in the last 6 months?” (0 = completely inactive to 10 = extremely active); “How active is your arthritis today with respect to joint tenderness and swelling?” (0 = completely inactive to 10 = extremely active); “How severe is your arthritis pain today?” (0 = no pain to 10 = unbearable pain); “How would you describe your general health today? (0 = very good to 10 = very bad); and “Did you experience joint (hand) stiffness on awaking yesterday morning? If yes, how long was this stiffness?” (0 = no stiffness to 10 = stiffness the whole day). In contrast, to the rather complicated formula of the original RADAI, the result can be easily calculated: (Q1 + Q2 + Q3 + Q4 + Q5)/5 [13]. For PsA patients, one question targeting patient’s assessment of skin involvement was added; “How active do you regard your skin disease? (0 = completely inactive to 10 = extremely active).

The intention behind the adaptation of RADAI-5 for assessment of PsA was that the five questions of the RADAI-5 cover five of six domains, being part of the OMERACT (Outcome Measures in Rheumatology) core set for PsA evaluation, namely joints, function, pain, patient’s global assessment, and quality of life [23], whereas the new sixth question explicitly targets the skin. We hypothesized that important domains of PsA activity assessment, such as enthesitis, dactylitis, and nail involvement are also at least in part covered by the questions targeting arthritis, pain and skin [24].

All patients gave their informed consent to be enrolled into this observational study, which was performed according to the Declaration of Helsinki. No objection against the study design was raised by the competent ethics committee, namely that of the Federal State of Lower Austria. Ninety-seven PsA outpatients (mean age 49.78 years; age range 23–80 years; 49 male, 48 female), all treated in a private rheumatology office or in a hospital-based outpatient clinic, were asked to complete the prototype questionnaire. Demographic details of these patients are given in Table 1.

**Table 1:** **Demographic data of patients completing the prototype six-item questionnaire**

**Patients**	**(n = 97)**	**Mean**	**Min**	**Max**
Gender (f/m)	48/49			
Caucasian ethnicity	100%			
Age (yrs.)		49.78	20.0	83.0
TJC		2.11	0	19
SJC		1.11	0	10
ESR		15.48	2	60
MDGlob		13.22	0	84
PatSat		2,48	1	5
DMARDs	75			
TNF-Blockers	12			

The tender joint count (TJC) and swollen joint count (SJC), including dactylitis and enthesiopathy (15), physician’s assessment of disease activity on a visual analog scale (VAS; 0–100; MDglob), erythrocyte sedimentation rate (ESR), and patient satisfaction with disease status (PatSat: 1 = excellent to 5 = unsatisfactory) were recorded [25]. Although not formally validated in PsA patients, PatSat was used to prove that the questionnaire really reflected the patient’s view of the disease; a basic requirement for any patient-related outcome measure.

### Statistical evaluation of the prototype questionnaire

Statistical evaluation was carried out using SPSS for Windows version 11.0. For internal consistency, assessment Cronbach’s alpha was calculated [26]. An alpha value of 0.70 indicates a standard error of measurement more than half (0.55) a standard deviation, therefore, higher values for individual assessments are necessary [27]. In addition, factor analysis by principal component analysis was performed to gain insight into the structure and item loading of the new questionnaire. To assess convergent validity by relating Stockerau Activity Score for Psoriatic Arthritis (SASPA) to ESR values, as well as SJC, TJC, and MDglob, Kendall’s tau was applied [28]. Its computation involves examining every pair of items and counting the number of pairs that are similarly ranked (concordant) and the number differently ranked (discordant) relative to each other on the two variables. The difference between the number of concordant and discordant pairs is divided by the total number of pairs. Tau values range from −1 (100% negative association, or perfect inversion) to +1 (100% positive association, or perfect agreement), and a value of 0 indicates the absence of association [28].

## Results

### Results for the prototype questionnaire

Cronbach’s alpha as a measure of internal consistency for the prototype six-item questionnaire was 0.861. Factorial analysis showed a mono-component structure (Eigenvalue 3.628) as well as an item loading between 0.558 (Question 6) and 0.892 (Question 3), indicating that every question contributed significantly to the aggregate result. Alpha was also calculated after deletion of one item, with the exception of Question 6, which was concerned with the severity of the skin disease. Item loading for Question 1 was 0.736, which appeared to be the lowest. As statistical reasons did not stand against and we wanted the new score to be as reliable as necessary, as parsimonious as possible and overall meeting the needs of practicability, the well-proven five-question principle and the simple calculation of RADAI-5 was retained [22].

As Question 6 is regarded as indispensable by all authors, subsequently, Question 1 from the RADAI-5 [22], targeting the course of the disease during the past 6 months, was removed from the questionnaire, which resulted in the highest alpha value of 0.849 for a five-item scale. Kendall’s tau as a measure of agreement between the six-item questionnaire and the version comprising five items was 0.908 (p < 0.001), indicating almost perfect agreement between the two scales.

### Analysis of the final questionnaire

The final five-item questionnaire, SASPA, (Table 2), was handed over to 152 PsA outpatients (mean age 54.02 years; age range 26–80 years; 82 male, 70 female), resulting in a total number of 779 completed questionnaires. All patients were treated in a private rheumatology office or in a hospital-based outpatient clinic. The demographic details of these patients are given in Table 3. In accordance with the analysis of the prototype questionnaire, TJC and SJC, including dactylitis and enthesiopathy [29], MDglob on a VAS (0–100), ESR (mm/h) as well as PatSat (1 = excellent to 5 = unsatisfactory) were recorded. The joint assessments during this observation were performed by three experienced physicians (BR, JS, and CD) in the outpatient department and exclusively by BFL in the private office. In order to avoid high internal variations among the physicians, consensus meetings concerning joint assessment are carried out at regular intervals as part of the routine quality control program of the outpatient department. For the purpose of this particular study, however, no formal agreement analysis between the physicians was performed.

**Table 2:** **SASPA**

**Question:**	**Range:**
How active is your arthritis today with respect to joint tenderness and swelling?”	(0 = completely inactive to 10 = extremely active)
“How severe is your arthritis pain today?”	(0 = no pain to 10 = unbearable pain)
“How would you describe your general health today?	(0 = very good to 10 = very bad)
“Did you experience joint (hand) stiffness on awaking yesterday morning? If yes, how long was this stiffness?”	(0 = no stiffness to 10 = stiffness the whole day)
“How active do you regard your skin disease?	(0 = completely inactive to 10 = extremely active)
**Final result: (Q1 + Q2 + Q3 + Q4 + Q5)/5**	**0 - 10**

**Table 3:** **Demographic data of patients completing SASPA**

**Patients**	**(n =152)**	**Mean**	**Min**	**Max**
Gender (f/m)	70/82			
Caucasian ethnicity	100%			
Age (yrs.)		54.02	26.0	80.0
TJC		1.92	0	28
SJC		1.24	0	10
ESR		14,828	1	90
MDGlob		8,6	0	65
PatSat		2.46	1	5
DMARDs	126			
TNF-Blockers	22			

### Evaluation of the final questionnaire

Statistical evaluation was carried out using SPSS for Windows version 11.0. For internal consistency, Cronbach’s alpha was calculated [26]. Fifty-three patients completed the SASPA questionnaire repetitively. The coefficient of variation, which estimated the percentage congruency of repeated measures, was calculated to avoid inclusion of redundant measurements [30]. In addition, factor analysis by principal component analysis was performed to gain insights into the structure and item loading of the new questionnaire. To assess convergent validity by relating the SASPA values to ESR values, as well as SJC, TJC, and MDglob, Kendall’s tau was applied [28]. For assessment of the sensitivity of the questionnaire to change, SASPA values before and after initiation of tumor necrosis factor (TNF) blocker were compared applying a paired *t* test. The respective standardized mean difference was calculated. The relationship between PatSat and the questionnaire was computed by analysis of variance (ANOVA).

### Results for the final questionnaire (SASPA)

The SASPA and PatSat values were normally distributed; therefore, the respective values are given as means and ranges. The mean SASPA was 2.66 (range: 0–9.2), and mean PatSat was 2.46 (range: 1–5) for the entire patient group. The coefficient of variation for repeated completions was 65.83%.

Cronbach’s alpha for the SASPA questionnaire was 0.875, easily surpassing the limit for substantial internal consistency in individual assessments. Factorial analysis by principal component analysis complied with the expectations and proved the one-dimensional structure of the questionnaire (Eigenvalue 3.424). Item loading ranged from 0.573 (Question 5) to 0.910 (Question 2), indicating that every item contributed significantly to the aggregate result.

**Figure 1:**
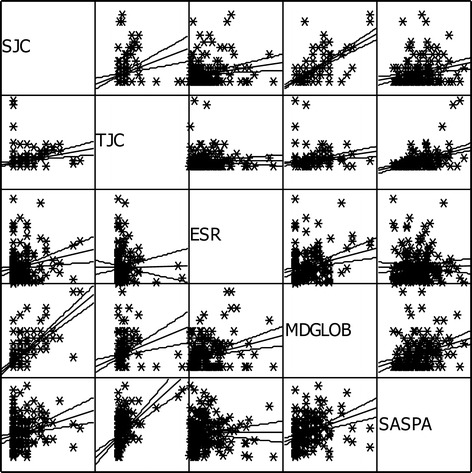
**Matrix-scattered plots to demonstrate the relationship between SASPA and TJC, SJC and MDglob.** Kendall’s tau for the relationship between SASPA results and SJC was 0.346 (p < 0.001), between SASPA and TJC, 0.416 (p < 0.001), between SASPSA and MDglob, 0.392 (p < 0.001), and between SASPA and ESR, 0.092 (n.s.).

After proving the internal consistency and analyzing the factorial structure, the relationship between SASPA values, derived from a questionnaire not including any joint count, and SJC, TJC, ESR, as well as MDglob, was important for assessing convergent validity. Kendall’s tau for the relationship between SASPA results and SJC was 0.346 (p < 0.001), and between SASPA and TJC it was 0.416 (p < 0.001). MDglob (tau 0.392; p < 0.001) was significantly related to SASPA values, while no significant relationship was found with ESR (tau 0.092) (Figure 1).

**Figure 2:**
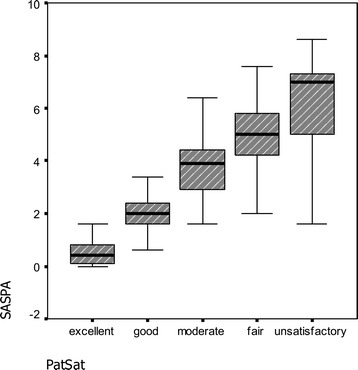
**Box-plot to demonstrate the relationship between PatSat and SASPA (p < 0.001 ANOVA, Bonferroni’s correction).**

ANOVA including Bonferroni’s correction showed a highly significant relationship between PatSat and SASPA levels (p < 0.001) (Figure 2).

### SASPA sensitivity to change

**Figure 3:**
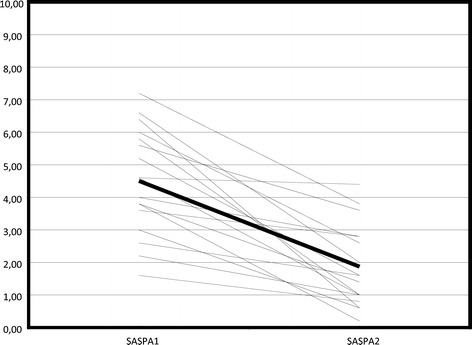
**Individual courses of SASPA in patients before (SASPA1) and after (SASPA2) initiation of TNF blockers.** The bold line denotes the average (standardized mean difference 2.1).

Nineteen patients (mean age 50.6 years; 12 male, 7 female) began TNF-blocker therapy during this observation. SASPA values before and a mean 4.1 months after treatment initiation were used to assess the sensitivity to change of the questionnaire. The mean SASPA at the start of TNF-blocker therapy was 4.51 (1.6–7.2), and after therapy it was 1.87 (0.2–4.4). This difference appeared to be highly significant according to a paired sample *t* test (p < 0.001). The respective standardized mean difference was 2.1, indicating a highly efficacious therapeutic intervention (Figure 3).

## Discussion

The main objective of this study was to assess whether a modified RADAI-5 questionnaire, namely SASPA, could be used as a fully patient-administered tool for daily PsA monitoring. SASPA should enable physicians to obtain reliable information about the disease course to support treatment decisions. It was not the intention with the development of SASPA, as with RADAI-5, to compensate for careful clinical examination [22]. Internal consistency, as a measure of the extent to which a variable or set of variables is stable, constitutes the primary prerequisite. Feasibility and acceptability to physicians and patients are also important requirements of any assessment tool. To cover the proposed core set measures for PsA as fully as possible, while keeping the balance between feasibility and reliability, it was decided to retain a five-question structure, as with RADAI-5 [24]. During all the studies to develop RADAI-5, support could be found for the arguments that RADAI-5 comprises questions targeting a patient’s pain perception and global health estimate, which can be seen as surrogates for functionality, and are likely also to include discomfort caused by enthesiopathy [22-24].

Self-report questionnaires, such as RADAI-5, can substitute for physician-derived disease activity scores, which were developed primarily for research purposes [19]. This may be expected highly likely also for SASPA in relation to PsA scores, such as DAPSA and PASDAS [11,12].

As with RADAI-5, SASPA allows better participation in the care of PsA patients, especially by non-specialists such as primary care physicians, without having to be trained in measuring formal joint counts.

PsA in contrast to RA often shows a highly variable course, including highly active as well as long-lasting inactive phases [1,2]. Nevertheless, PsA may cause severe joint destruction and functional loss in some patients [2]. From the patient’s position, PsA may cause joint problems, as well as skin manifestations, particularly of the nails. Moreover, and, of course individually aggravating, the disease burden’s focus may shift from the musculoskeletal system to the skin or vice versa [31]. Therefore, we are convinced that rheumatologists and dermatologists should pay attention to the skin and joint manifestations before deciding on treatment [32]. This was why we insisted on including the question targeting the severity of the skin manifestations. These considerations were the basis for the ultimate decision to leave out the question targeting the course of the disease during the past 6 months.

With regard to physician- or patient-reported outcome measures, SASPA, as for RADAI-5, can be regarded as a hybrid tool, namely, a tool assessed by patients, comprising domains selected by physicians. Patients agreed that the SASPA questionnaire was easy and rapid to complete. SASPA has the advantage that the individual patient – the primary target of all therapeutic interventions – is given the key role with respect to disease activity assessment. Beyond that, inter-physician, but also intra-physician variations in assessing joints or global disease activity are eliminated, and other pitfalls of joint counts are avoided [33]. The lower the number of involved joints, the more intra- and inter-observer variance exerts an influence upon relative assessment tools, which is of particular importance in PsA, because it constitutes an oligoarticular disorder in some patients [1,2,33].

Measurement of patient symptoms alone may have an important shortcoming, namely that symptom levels are differently evaluated and expressed among patients. However, physical function on a health assessment questionnaire, the most frequent patient-reported outcome, is the measure that is most significant in identifying and predicting work disability, explaining costs, and predicting mortality in RA – much more effectively than the other core data set measures [34].

The newly adapted SASPA was investigated for its psychometric properties. Cronbach’s alpha was >0.8, indicating high internal consistency. Moreover, the questionnaire proved to be a one-dimensional instrument with some differences in item weighing, with the question targeting the severity of skin disease showing the lowest one. One reason for that could be that PsA patients in our unit have a highly variable but generally mild psoriatic skin disease, and primarily seek to reduce the intensity of their joint complaints.

SASPA was shown to be in moderate agreement, however significantly correlated, with most of the ACR core set measures such as TJC, SJC, and MDglob [22,35]. This on the one hand can be regarded as proof of convergent validity, and on the other hand, also as a possibility to eliminate difficulties with joint counts in daily routine rheumatology [33]. The relationships obtained here are in line with the results for RADAI-5. One of the reasons for this moderate agreement may be differences in patients’ weightings of joint involvement and physicians’ joint counts. SASPA includes two questions that target all joints and entheses as a whole, enabling the patients to weigh their individual burden, which is not possible if single joints are simply counted, and identically weighted, for example, the fifth metacarpal joint being treated in the same manner as the knee.

PsA activity assessment without joint counts has the advantage of not losing activity by applying certain counting models. In contrast to RA, no consensus has yet been reached by which counting models can be applied in daily routine assessment of individual PsA patients [36]. The 28-joint count e.g. excludes the foot joints, which cannot be regarded appropriate in a disease frequently presenting with an oligoarticular pattern, and involving the lower extremities. Such joint counting models may lead to misclassification of some patients [1,2,33]. To refrain from routine joint counts does not mean disregarding joint examination, but rather giving a place for focused patient evaluation. Nevertheless, as individualized treatment becomes increasingly important, patient-related outcome tools could provide substantial advantages in identifying patients requiring particular attention.

The sensitivity to change of the questionnaire was tested in a subgroup of patients starting TNF-blocker therapy. SASPA was highly sensitive to change, resulting in a standardized mean difference for the particular intervention indicating high efficacy. This study was performed in a routine clinical setting, therefore, we took initiation of TNF-blocker as the standardized therapeutic intervention and ≥2 months thereafter as the decisive time point to assess efficacy.

The present study had several limitations. First, the study was performed in two closely related offices within a relatively small region. Second, increased self-efficacy, as a member of a study population, constitutes an important factor possibly influencing patient’s self- assessment [37]. Third, patients included in this observation did not have severe skin manifestations. SASPA should be compared to composite indexes for PsA. We are confident that the respective results will be in agreement with those obtained for RADAI-5 and RAPID 3 in RA patients.

However, as with the other instruments, only stable low SASPA values can be regarded as indicators of an uncomplicated disease course [38]. Significant changes, however, must be appraised with respect to the changes in the single items and possible coexisting or newly occurring diseases [39]. Our results provide robust evidence that patient-centered disease activity assessment is reliable and feasible for daily routine monitoring of PsA, as in RA or ankylosing spondylitis [22,40].

## Conclusions

During this observational study, we demonstrated that SASPA, excluding joint counts, could measure PsA activity and was sensitive to change. We also demonstrated the reliability and convergent validity of this questionnaire, derived from RADAI-5. Therefore, we propose the SASPA questionnaire as an option for routine monitoring of PsA patients, enabling physicians to obtain reliable information about disease course and thereby providing support in treatment decisions.
